# Chronic circadian misalignment results in reduced longevity and large-scale changes in gene expression in *Drosophila*

**DOI:** 10.1186/s12864-018-5401-7

**Published:** 2019-01-07

**Authors:** Alex C. Boomgarden, Gabriel D. Sagewalker, Aashaka C. Shah, Sarah D. Haider, Pramathini Patel, Heather E. Wheeler, Christine M. Dubowy, Daniel J. Cavanaugh

**Affiliations:** 1 Department of Biology, Loyola University Chicago, 1050 W. Sheridan Rd, Chicago, IL 60660 USA; 2 Department of Computer Science, Loyola University, Chicago, 60660 USA; 3 Department of Psychiatry, University of Pennsylvania Perelman School of Medicine, Philadelphia, PA 19104 USA

**Keywords:** Drosophila, Circadian misalignment, Longevity, RNA-sequencing

## Abstract

**Background:**

Circadian clocks are found in nearly all organisms, from bacteria to mammals, and ensure that behavioral and physiological processes occur at optimal times of day and in the correct temporal order. It is becoming increasingly clear that chronic circadian misalignment (CCM), such as occurs in shift workers or as a result of aberrant sleeping and eating schedules common to modern society, has profound metabolic and cognitive consequences, but the proximate mechanisms connecting CCM with reduced organismal health are unknown. Furthermore, it has been difficult to disentangle whether the health effects are directly induced by misalignment or are secondary to the alterations in sleep and activity levels that commonly occur with CCM. Here, we investigated the consequences of CCM in the powerful model system of the fruit fly, *Drosophila melanogaster*. We subjected flies to daily 4-h phase delays in the light-dark schedule and used the *Drosophila* Activity Monitoring (DAM) system to continuously track locomotor activity and sleep while simultaneously monitoring fly lifespan.

**Results:**

Consistent with previous results, we find that exposing flies to CCM leads to a ~ 15% reduction in median lifespan in both male and female flies. Importantly, we demonstrate that the reduced longevity occurs independent of changes in overall sleep or activity. To uncover potential molecular mechanisms of CCM-induced reduction in lifespan, we conducted whole body RNA-sequencing to assess differences in gene transcription between control and misaligned flies. CCM caused progressive, large-scale changes in gene expression characterized by upregulation of genes involved in response to toxic substances, aging and oxidative stress, and downregulation of genes involved in regulation of development and differentiation, gene expression and biosynthesis.

**Conclusions:**

Many of these gene expression changes mimic those that occur during natural aging, consistent with the idea that CCM results in premature organismal decline, however, we found that genes involved in lipid metabolism are overrepresented among those that are differentially regulated by CCM and aging. This category of genes is also among the earliest to exhibit CCM-induced changes in expression, thus highlighting altered lipid metabolism as a potentially important mediator of the negative health consequences of CCM.

**Electronic supplementary material:**

The online version of this article (10.1186/s12864-018-5401-7) contains supplementary material, which is available to authorized users.

## Background

The daily rotation of the Earth on its axis produces predictable cycles of light and temperature. This has led to the evolution of endogenous circadian clocks, which allow organisms to anticipate and adapt to environmental cycles to ensure that behavioral and physiological processes occur at optimal times of day. Perhaps equally importantly, circadian clocks orchestrate the temporal sequencing of internal events such that they occur at appropriate times with respect to one another. Together, these extrinsic and intrinsic advantages of circadian clocks are thought to promote organismal health [1]. For example, the circadian system generates rhythms in foraging and feeding behaviors such that they occur during times of maximal food availability, and concurrently upregulates metabolic pathways in anticipation of increased food intake.

Research undertaken over the past ~ 50 years has identified many of the genes and molecules that confer upon cells the ability to keep time. In most organisms, the molecular circadian clock consists of a transcriptional-translational feedback loop that produces rhythmic oscillations in gene expression, and this molecular clock is present both in master circadian pacemaker cells in the brain as well as in cells in peripheral tissues [2, 3]. A key feature of circadian clocks is their ability to maintain rhythmic oscillations under constant conditions; however, because they oscillate with an intrinsic period that is not exactly 24-h, they must be reset on a daily basis through the process of entrainment in order to remain synchronized to environmental cues [4]. More extensive clock resetting is necessary following abrupt shifts in lighting schedules, such as occur with trans-meridian travel, shiftwork, or simply as a result of the aberrant eating and sleeping schedules that are common in modern society. The latter in particular, which has been termed ‘social jetlag’, has led to a situation in which as much as 2/3 of the population live under a state of chronic circadian misalignment (CCM) typified by a behavioral schedule that runs outside the dictates of their endogenous circadian rhythms [5, 6]. These irregular schedules likely result in a misalignment between circadian clocks and external environmental signals as well as internal desynchrony between different clock-containing tissues in the body.

Evidence suggests that circadian disruption has severe consequences. Epidemiological studies in humans have demonstrated that various forms of CCM are correlated with higher incidences of a number of disorders including obesity, diabetes, cardiovascular disease, cancer, and mood disorders, along with increased overall mortality rates. [4, 7, 8]. Controlled studies using animal models have corroborated these findings and demonstrated a causal link between CCM and decreased health and longevity, both in invertebrate and vertebrate systems [7–17]. However, despite a growing knowledge of the importance of circadian function for organismal health, the cellular and molecular mechanisms through which CCM negatively impacts health and longevity have yet to be established. Furthermore, it is unclear whether the negative health effects are directly caused by misalignment or whether they are secondary to behavioral changes, such as reduced sleep, that often accompany CCM [8].

Here we employed a model of CCM in the fruit fly, *Drosophila melanogaster*, in which we exposed flies to daily 4-h phase delays in lighting conditions. We used the Drosophila Activity Monitoring (DAM) system to continuously monitor fly locomotor activity and sleep while simultaneously conducting longevity analysis. This allowed us to assess the effect of long-term CCM on aging-associated changes in locomotor activity and sleep levels, and to correlate these measures with fly lifespan. We demonstrate that CCM leads to an ~ 15% reduction in median lifespan of both male and female flies, in agreement with previous studies [10, 12]. While misaligned flies exhibited aberrant patterns of locomotor activity, as evidenced by reduced rest:activity rhythm strength under light-dark (LD) conditions, overall sleep and activity levels were largely unchanged and did not account for the reduction in longevity produced by CCM. We further conducted RNA sequencing analysis and identified several categories of genes that are up- or downregulated by CCM. Interestingly, many of the gene expression changes induced by CCM mirror those observed with natural aging, suggesting that CCM results in cellular stress and premature organismal decline. However, we find that genes involved in lipid metabolism, which are among the earliest to undergo CCM-induced changes in expression, are also differentially regulated by CCM and aging, highlighting this category of genes as a potentially important mediator of the negative consequences of circadian disruption.

## Results

### CCM reduces lifespan

**Fig. 1:**
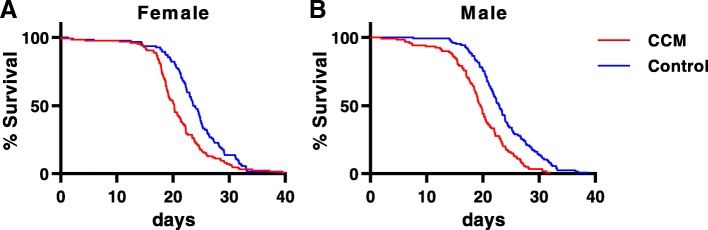
CCM reduces fly longevity. Percent of female (**a**) and male (**b**) flies surviving during exposure to either a control (blue) or a CCM (red) schedule over the course of a 40-day experiment. Dotted lines indicate 95% confidence intervals. CCM decreased longevity for both female (14.8% reduction in median lifespan, *p* = 1.56e-04; Log-rank test; *n* = 124 for control and 126 for CCM) and male (14.7% reduction in median lifespan, *p* = 2.65e-07, Log-rank test; n = 125 for control and 120 for CCM) flies

We initially assessed fly longevity to determine overall health consequences of CCM. Consistent with previous results [10, 12], we found that exposure to daily 4-h phase delays in lighting conditions significantly reduced lifespan in both male and female flies (Fig. 1a and b). Thus, median lifespan was 23.0 and 23.7 days for control males and females, respectively, and this was reduced to 19.6 days for CCM-exposed males and 20.2 days for CCM-exposed females. This represents an equivalent reduction of median lifespan by 14.8% in females and 14.7% in males.

It should be noted that all flies were maintained on minimal sucrose-agar food during longevity analysis, resulting in a relatively short lifespan of even control flies in these experiments compared with those performed with standard food. This food source is typical for use with the DAM system and thus allowed us to more directly compare activity and sleep levels in our experiments to those observed in other studies using this system. Nevertheless, the ~ 15% reduction in median lifespan we observed agrees well with results obtained with flies kept on standard food [10], which demonstrates that the negative health impacts of CCM are similar regardless of diet.

### CCM causes aberrant locomotor activity patterns

**Fig. 2:**
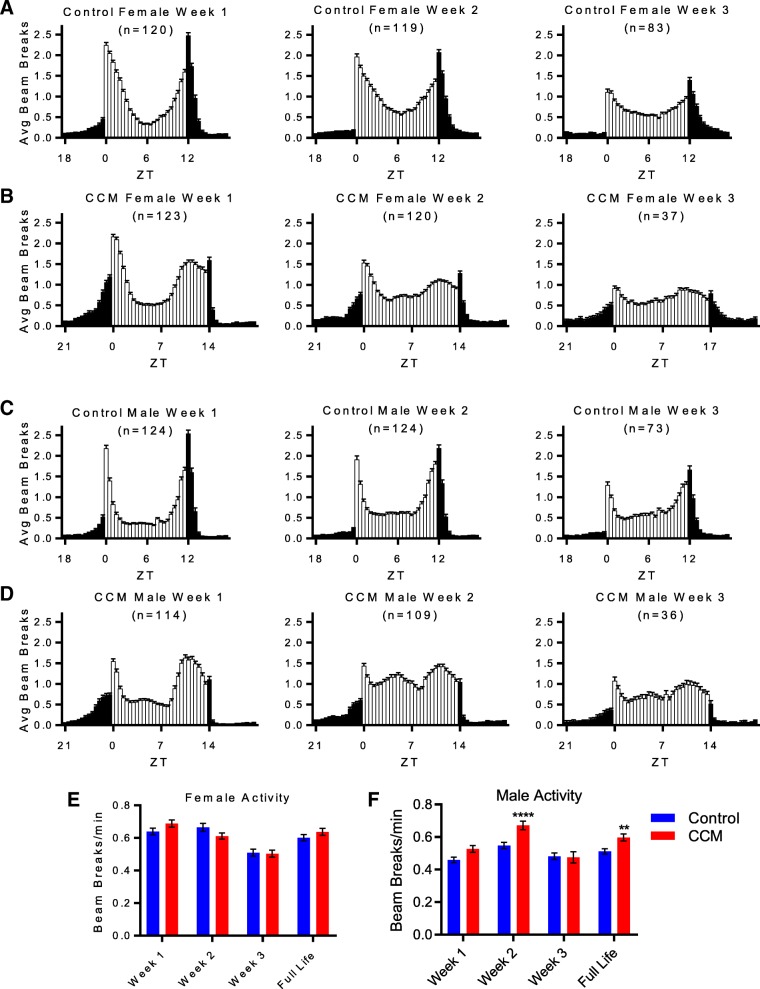
CCM causes aberrant locomotor activity behavior. **a**-**d** Average activity profiles for the indicated groups and time points depict the number of DAM beam breaks/min in 30 min bins for an entire light-dark cycle (which consisted of 24 h for controls and 28 h for CCM flies) averaged across one week (seven 24-h days for control or six 28-h days for CCM flies). White and black bars correspond to light and dark periods, respectively. Error bars represent standard error of the mean (SEM). ZT refers to zeitgeber time, with ZT0 being the time of lights-on. **e**-**f** Weekly activity levels in mean beam breaks/min ± SEM are shown for female (**e**) and male (**f**) flies for weeks 1, 2 and 3 and across the full life of the animals. For females, 2-Way ANOVA indicated a significant effect of time (F_(3, 869)_ = 15.1, *p* = 1.47E-9) but not treatment (F_(1, 869)_ = 0.149, *p* = 0.700) and Sidak’s posthoc test revealed no differences between control and CCM-exposed flies for any time point (*p* ≥ 0.237 for all). For males, 2-Way ANOVA indicated a significant effect of time (F_(3, 831)_ = 15.2, *p* = 1.25E-9) and treatment F_(1, 831)_ = 17.9, *p* = 2.61E-5) and Sidak’s posthoc test revealed a significant difference between control and CCM-exposed flies for week 2 (*p* = 6.97E-5) and full life (*p* = 8.30E-3) but not week 1 (*p* = 0.063) or week 3 (*p* = 0.999)

**Fig. 3:**
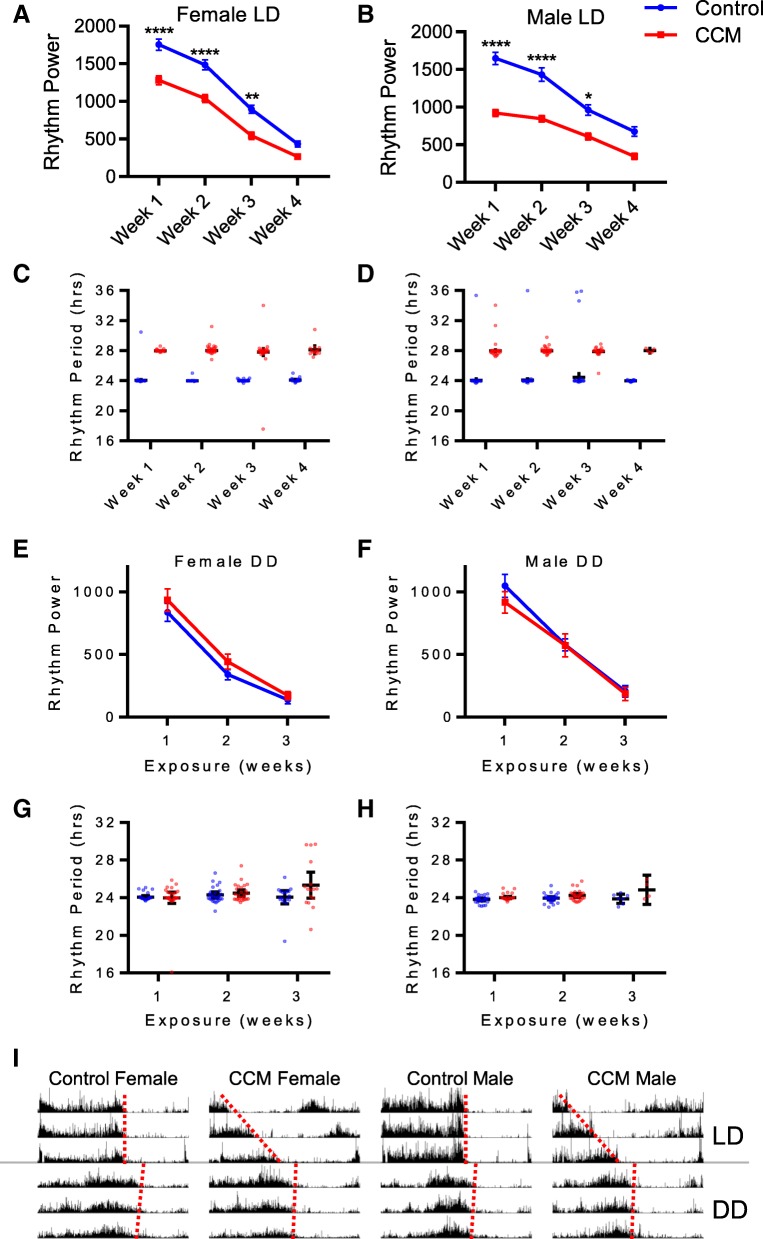
CCM reduces LD rest:activity rhythm strength but does not affect free-running rhythms. **a**-**b** Mean rest:activity rhythm power ± SEM is shown for female (**a**) and male (**b**) flies for each week of the experiment during exposure to control (blue) or CCM (red) conditions. For female flies, 2-Way ANOVA indicated a significant main effect of time (F_(3, 651)_ = 66.7, *p* < 1.0E-15) and treatment (F_(1, 651)_ = 28.8, *p* = 1.09E-7) and Sidak’s posthoc test revealed significant differences between control and CCM females for weeks 1 (*p* = 3.72E-8), 2 (*p* = 4.47E-7) and 3 (*p* = 6.73E-3), but not for week 4 (*p* = 0.893). Similar results were observed in male flies, for which 2-Way ANOVA indicated a significant main effect of time (F_(3, 611)_ = 16.7, *p* = 1.90E-10) and treatment (F_(1, 611)_ = 25.6, *p* = 5.45E-7), and Sidak’s posthoc test revealed significant differences between control and CCM males for weeks 1 (*p* = 4.20E-14), 2 (*p* = 3.20E-9) and 3 (*p* = 3.39E-2), but not for week 4 (*p* = 0.813). For all graphs, ****p < 0.0001; **p < 0.001; *p < 0.01, Sidak’s posthoc test. **c**-**d** Rest:activity rhythm period is shown for female (**c**) and male (**d**) flies for each week of the experiment during exposure to control (blue) or CCM (red) conditions. Each dot represents the period of an individual fly and lines indicate mean ± 95% confidence intervals. (**e**-**f**) Mean free-running rest:activity rhythm power ± SEM is shown for female (**e**) and male (**f**) flies previously exposed to 1, 2 or 3 weeks of control (blue) or CCM (red) conditions. For females, 2-Way ANOVA indicated a significant effect of time (F_(2, 138)_ = 59.1, *p* < 1.0E-15) but not treatment (F_(1, 138)_ = 1.91, *p* = 0.169). Similarly, for males, 2-Way ANOVA indicated a significant effect of time (F_(2, 119)_ = 22.7, *p* = 4.54E-9) but not treatment (F_(1, 119)_ = 0.255, *p* = 0.614). **g**-**h** Free-running rest:activity rhythm period is shown female (**g**) and male (**h**) flies previously exposed to 1, 2 or 3 weeks of control (blue) or CCM (red) conditions. Each dot represents the period of an individual fly and lines indicate mean ± 95% confidence intervals. For E and G, *n* = 28 for 1-week control females, *n* = 30 for 1-week CCM-exposed females, *n* = 26 for 2-week control females, *n* = 27 for 2-week CCM-exposed females, *n* = 17 for 3-week control females, and *n* = 16 for 3-week CCM-exposed females. For F and H, n = 30 for 1-week control males, n = 30 for 1-week CCM-exposed males, n = 28 for 2-week control males, n = 26 for 2-week CCM-exposed males, *n* = 7 for 3-week control males, and *n* = 4 for 3-week CCM-exposed males. **i** Mean normalized activity is plotted for the last three days in LD followed by the first three days in DD for the genotypes listed. Flies were transferred to DD following 2 weeks exposure to CCM or control conditions. Data are single plotted and each line is 24 h. Dotted red lines track activity offset. n = 16 per group. Note the immediate transition to ~ 24-h free-running periods for all groups after transfer to DD

Our use of the DAM system to monitor locomotor activity throughout the duration of the longevity experiment allowed us to determine whether activity patterns were altered by CCM exposure. At the gross behavioral level, we found evidence that flies were able to entrain to the constantly shifting lighting schedule. The daily 4-h phase delays produce a 28-h LD schedule, and flies exposed to such conditions exhibited behavioral rhythms with 28 h periods (Fig. 2; Fig. 3c-d). This corroborates similar findings in blowflies exposed to 27 h days [11] and supports the idea that flies have wide limits of entrainment [18]. It is also possible; however, that the apparent entrainment to aberrant lighting schedules that others and we have observed is actually due to a masking effect of light exposure directly driving fly activity.

Despite the fact that rest:activity rhythms remained time-locked to phase-delaying schedules, we did observe activity patterns in CCM-exposed flies indicative of mistimed locomotor activity. In flies, dawn and dusk lighting transitions are normally preceded by anticipatory increases in locomotor activity that depend on a functional circadian clock [3]. In young control flies, we found that morning and evening anticipatory activity began ~ 8–9 h following the previous lighting transition, with more prominent activity increases occurring in anticipation of lights turning off (Fig. 2a and c). CCM flies also began to ramp up their activity ~ 8–9 h following the previous lighting transition; however, because of the delayed lighting schedule, this led to a substantial mistiming of anticipatory activity which resulted in prolonged activity peaks that started to resolve prior to the actual lighting transitions (Fig. 2b and d). This mistimed anticipation of lighting conditions persisted throughout the duration of the experiment. Thus, in weeks 2 and 3, although all flies displayed lower amplitude activity rhythms, CCM-exposed flies continued to show prolonged activity peaks that preceded lighting transitions by several hours more than controls (Fig. 2a-d). In addition to this prolonged anticipatory activity preceding lighting changes, CCM flies exhibited a modest mid-afternoon activity peak during the normal siesta time. This was most prominently observed in males during the second week of CCM exposure (Fig. 2d). This increased daytime activity may account in part for the increase in week 2 and lifetime activity levels in male CCM flies compared to controls. In contrast, overall activity was unchanged in CCM females (Fig. 2e-f). Together, these results demonstrate that circadian clocks in CCM-exposed flies have to be constantly adjusted to changing environmental cycles, leading to chronically mistimed activity.

To determine whether CCM affects rhythm amplitude in addition to timing of activity, we conducted chi^2^ periodogram analysis, which demonstrated that CCM-exposed flies displayed reduced locomotor activity rhythm strength compared to controls (Fig. 3a and b). Both experimental and control flies showed expected reductions in rest:activity rhythm strength as they aged [19] (Fig. 3a-b), typified by less robust activity peaks and reduced anticipatory activity, including an apparent lack of lights-on anticipation in control flies by week 3 (Fig. 2a and c). However, CCM flies had lower rhythm power than controls at all time points, perhaps as a result of mistimed morning and evening anticipation relative to lights-on and lights-off, leading to prolonged activity bouts.

This reduced rhythmicity could be due either to the misalignment between internal and external rhythms or to CCM-induced damage to core clock neurons or molecular cycling. To test for the latter, we assessed locomotor rhythmicity of flies in constant darkness (DD) following exposure to varying amounts of time in either CCM or control conditions (Fig. 3e-h). Importantly, we saw no differences in free-running rhythm power or period between control and experimental flies. Furthermore, CCM flies immediately adopted normal free-running periods upon transition to DD conditions, showing no residual effect of their prolonged 28-h day exposure (Fig. 3i). The fact that CCM flies have normal free-running rhythms indicates that the central clock and associated output pathways maintain proper function following CCM exposure and further suggests that the reduced rhythm strength observed under LD conditions is a result of the discrepancy between the endogenous period of the fly and environmental cycles.

### CCM decreases longevity independent of changes in locomotor activity or sleep

**Fig. 4:**
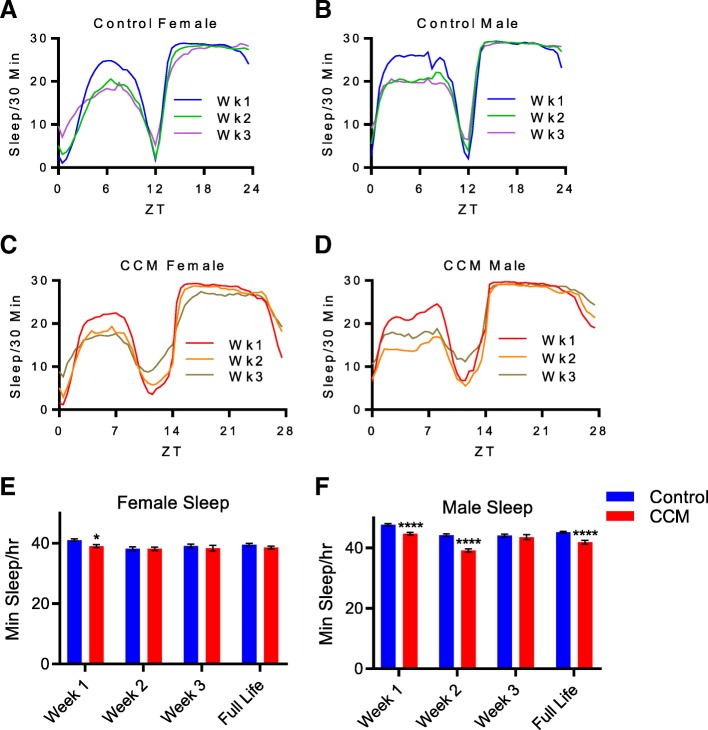
CCM causes minor reductions in sleep duration. **a**-**d** Mean sleep/30 min ± SEM is shown for female (**a** and **c**) and male (**b** and **d**) flies over the course of a single light-dark cycle averaged over one week. For control flies (**a** and **b**), an average 24-h day is shown, blue bars depict sleep during week 1, green bars depict sleep during week 2 and magenta bars depict sleep during week 3. For CCM-exposed flies (**c** and **d**), an average 28-h day is shown, red bars depict sleep during week 1, orange bars depict sleep during week 2 and brown bars depict sleep during week 3. **e**-**f** Mean min sleep/hr. ± SEM is shown for female (**e**) and male (**f**) flies for weeks 1, 2 and 3 and across the full life of the animals during exposure to control (blue) or CCM (red) conditions. For females, 2-Way ANOVA indicated a significant effect of treatment (F_(1, 866)_ = 5.17, *p* = 2.32E-2) and time (F_(3, 866)_ = 4.38, *p* = 4.52E-3) and Sidak’s posthoc test revealed a significant difference between control and CCM-exposed flies for week 1 (*p* = 2.81E-2), but not for week 2 (*p* = 0.999), week 3 (*p* = 0.919) or full life (*p* = 0.584). For males, 2-Way ANOVA indicated a significant effect of treatment (F_(1, 828)_ = 63.5, *p* < 1.0E-15) and time (F_(3, 828)_ = 31.4, *p* = 5.0E-15) and Sidak’s posthoc test revealed a significant difference between control and CCM-exposed flies for week 1 (*p* = 2.3E-5), week 2 (*p* = 3.39E-13), and full life (*p* = 1.96E-6) but not week 3 (*p* = 0.973)

The mechanisms through which chronic misalignment affects physiological health have yet to be clearly defined; however, one possibility is that CCM indirectly decreases health secondary to alterations in activity levels or sleep duration [8]. To determine whether alterations in sleep could account for the reduced longevity we observed in our experiments, we first analyzed locomotor activity data to determine if sleep is altered by CCM. In general, sleep patterns appeared normal in CCM-exposed flies. Like controls, experimental flies had a consolidated period of sleep at night and an additional siesta period of sleep in the middle of the afternoon (Fig. 4a-d). However, consistent with our activity rhythm observations, we found that CCM-exposed flies had prolonged periods of suppressed sleep prior to lighting transitions. In agreement with other reports [19–21], we also noted that in later weeks of the experiment, both control and experimental flies experienced a decrease in daytime sleep and a reduction in sleep rhythm amplitude characterized by increased sleep duration during lighting transitions (Fig. 4a-d). Furthermore, both control and CCM-exposed male flies consistently slept more than their female counterparts (Fig. 4e-f), which is typical for *Drosophila* [22].

**Fig. 5:**
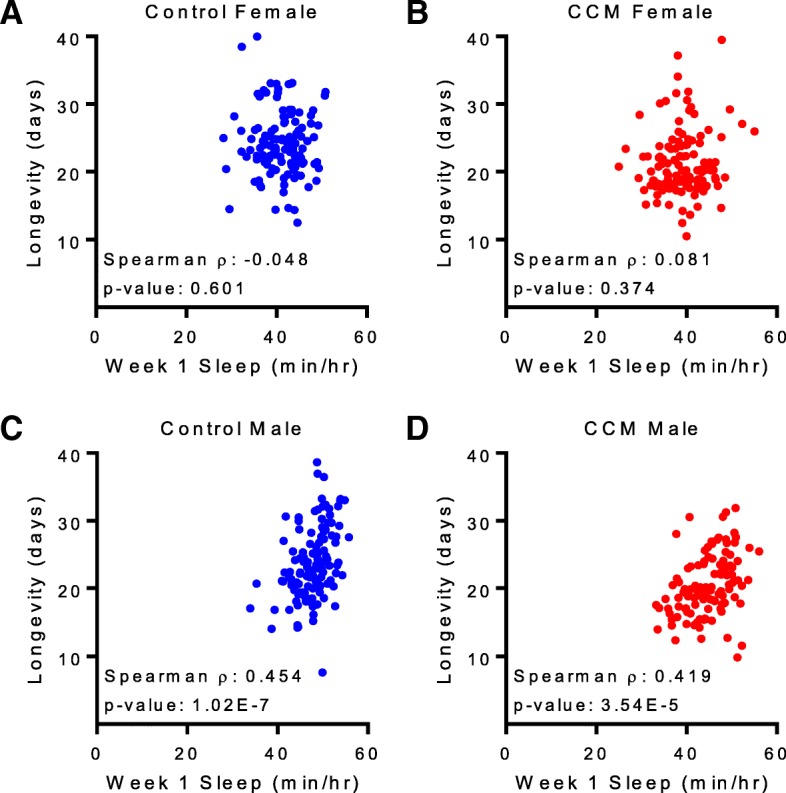
Baseline sleep duration correlates with longevity in male flies. **a**-**d** Mean sleep/hour during the first week of life is plotted against lifespan for flies exposed to either control (**a** and **c**; blue) or CCM (**b** and **d**; red) conditions. Each dot represents an individual fly. While female flies (**a** and **b**) showed no relationship, both control (**c**) and CCM-exposed (**d**) males showed significant, positive correlations between early life sleep and longevity. For control females, *n* = 121; Spearman rho = − 0.048 (95% confidence interval: − 0.230 to 0.137); *p* = 0.601. For CCM female, *n* = 123; Spearman rho = − 0.081 (95% confidence interval: − 0.103 to 0.259); *p* = 0.374. For control males, *n* = 125; Spearman rho = 0.454 (95% confidence interval: 0.249 to 0.563); *p* = 1.02E-7. For CCM males, *n* = 114; Spearman rho = 0.419 (95% confidence interval: 0.298 to 0.587); *p* = 3.54E-5

Despite the fact that sleep architecture was generally unchanged, we found that both male and female flies exposed to CCM exhibited a minor yet significant reduction in sleep amount during the first week of behavioral monitoring (Fig. 4e-f). In males, this sleep reduction persisted into the second week and also manifested as a decrease in average sleep duration across the full life of the animals (Fig. 4f). Despite these differences, it is important to note that CCM flies still obtain a substantial amount of daily sleep, with male and female CCM-exposed flies getting ~ 93 and 95% of control levels, respectively. Nevertheless, because CCM resulted in significantly reduced sleep, it was possible that this accounted for the decreased longevity in CCM-exposed flies, especially since we found that in males, average daily sleep duration in the first week of life correlated with longevity (Fig. 5c-d). Similar findings have been reported previously [23, 24]. Week 2 sleep duration was also correlated with longevity in males, but this correlation was lost by week 3, suggesting that early life sleep is particularly impactful for lifespan determination. Interestingly, this relationship did not hold for females (Fig. 5a-b), which to our knowledge have not previously been tested for such a correlation.

To further assess the effect of CCM and sleep on longevity, we used the Cox Proportional Hazard model. We first calculated hazard ratios for CCM and sleep time in univariate models. The hazard ratio is the ratio of the chance of death in one condition to the chance of death in the other condition at any point in time. If the hazard ratio is one, there is no difference in the rate of death between groups. A hazard ratio less than one indicates a beneficial effect on survival, while a hazard ratio more than one indicates a detrimental effect on survival. In male flies, the estimated hazard ratio for CCM in a univariate model is 1.94 (Table 1), indicating that CCM-exposed flies are 94% more likely to die at any point in time compared to control flies. The hazard ratio for week 1 sleep time in a univariate model is 0.74 (Table 1), indicating that for each additional hour of daily sleep a fly is 26% less likely to die at a given point.

**Table 1:** Effect of CCM and Sleep on Survival

Males			
Effect of CCM on Survival in Univariate Cox Proportional Hazard Model			
Variable	Hazard Ratio	Confidence Interval	Statistical Significance
CCM	1.94	1.49–2.53	*p* < 0.001 ***
Effect of Sleep Time on Survival in Univariate Cox Proportional Hazard Model			
Variable	Hazard Ratio	Confidence Interval	Statistical Significance
Sleep (Per Hour)	0.74	0.69–0.80	*p* < 0.001 ***
Effect of CCM and Sleep Time on Survival in Multivariate Cox Proportional Hazard Model			
Variable	Hazard Ratio	Confidence Interval	Statistical Significance
CCM	1.49	1.13–1.98	*p* < 0.01 **
Sleep (Per Hour)	0.77	0.71–0.83	*p* < 0.001 ***
Effect of CCM on Survival in Sleep-Matched Univariate Cox Proportional Hazard Model			
Variable	Hazard Ratio	Confidence Interval	Statistical Significance
CCM	1.37	1.02–1.85	*p* < 0.05 *
Females			
Effect of CCM on Survival in Univariate Cox Proportional Hazard Model			
Variable	Hazard Ratio	Confidence Interval	Statistical Significance
CCM	1.65	1.28–2.12	*p* < 0.001 ***
Effect of Sleep Time on Survival in Univariate Cox Proportional Hazard Model			
Variable	Hazard Ratio	Confidence Interval	Statistical Significance
Sleep (Per Hour)	0.97	0.91–1.03	*p* = 0.32

Because CCM shortens daily sleep time, we wanted to know if the effect of CCM on survival was due to the shortened sleep of these flies or if CCM was more detrimental than would be predicted by its effects on sleep time alone. We took two approaches. In one approach, we used a multivariate Cox Proportional Hazard Model with both treatment and week 1 sleep time in the model. The hazard ratio for CCM in the multivariate model was 1.49 (Table 1), indicating that misaligned flies were roughly 49% more likely to die than controls flies at a given point in time, even with sleep time held constant. Put another way, the risk of death for a CCM fly was roughly equivalent to the risk of death for a control fly with an hour and a half less sleep. In the other approach, we matched each control fly with a CCM fly with a similar sleep time and calculated a hazard ratio for CCM in this smaller, sleep-matched data set, which importantly also normalized overall activity levels in CCM flies. This produced a similar result, with a hazard ratio of 1.37 for CCM (Table 1). In female flies, the estimated hazard ratio for CCM in a univariate model was 1.65 (Table 1), although we note that the model assumption of proportional hazards is not upheld for female flies, as CCM has a stronger effect on survival at earlier time points. Sleep time did not significantly affect survival in female flies, so was not a potential confounding factor. It is therefore clear that the effect of CCM on survival in female flies is not due to its effects on sleep time. Together, these analyses demonstrate that the reduced longevity resulting from CCM is independent of the minor changes in activity and sleep that are associated with the daily 4-h phase delay schedule. This idea is further supported by the fact that CCM equivalently reduced lifespan in male and female flies (Fig. 1a and b) despite the fact that males and females have differences in baseline sleep and that CCM more strongly reduced sleep amount in males than females (Fig. 4e and f).

### CCM induces large-scale changes in gene expression

Having determined that CCM reduces lifespan independent of overt behavioral changes, we sought to identify changes at the molecular level that could underlie the physiological consequences of CCM. We therefore used whole-fly RNA sequencing to assess changes in gene transcription associated with prolonged circadian misalignment. For these experiments, we pooled male and female flies, which we reasoned would allow us to identify central, common mechanisms through which circadian misalignment reduces lifespan, even though this may obscure sex-specific gene expression changes.

**Fig. 6:**
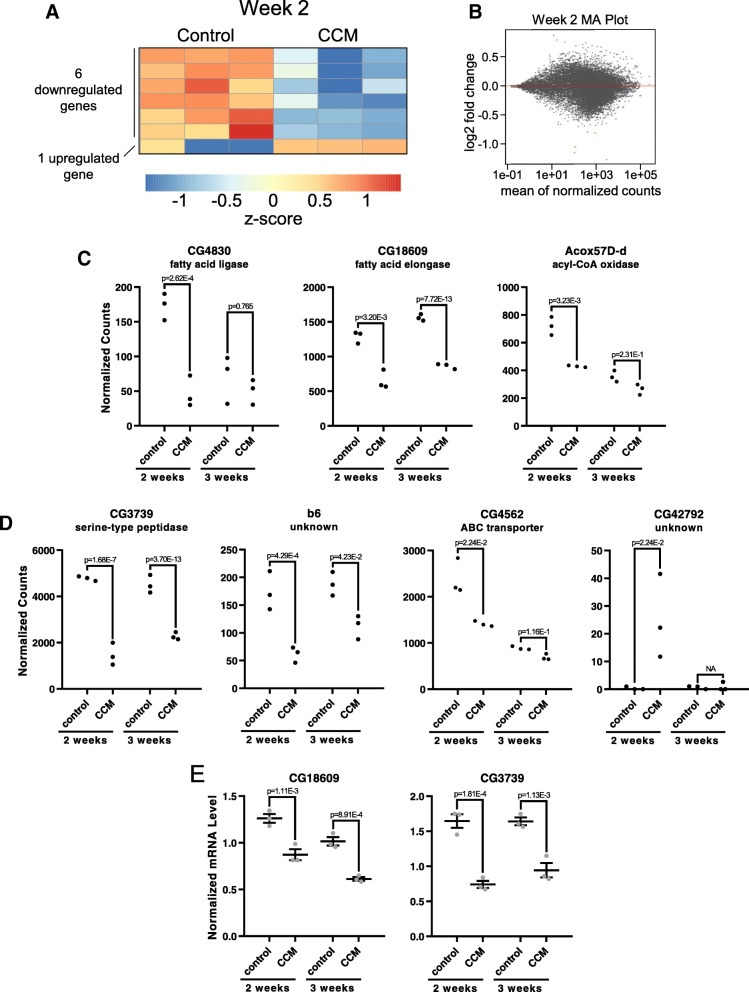
Gene expression changes following 2 weeks of CCM exposure. **a** Heatmap showing z-score of DEseq2 normalized counts for all genes found to be differentially expressed (at a FDR < 0.1) following 2 weeks of CCM or control conditions. Each condition includes 3 biological replicates. **b** MA plot for week 2 differential expression analysis. Log2 fold change (M) is plotted against average normalized gene expression (A). Red dots indicate differentially expressed genes. **c** Expression plots showing normalized gene expression levels for week 2 differentially expressed genes annotated with lipid metabolism functions. Expression levels are shown for both week 2 and week 3 differential expression analysis. Dots represent individual samples. Note that not all genes had statistically significant week 3 changes. Benjamini-Hochberg corrected *p* values are based on DEseq2 analysis. **d** Expression plots for other week 2 differentially expressed genes are graphed as in C. NA indicates that the gene was filtered out during DEseq2 analysis due to low count number. **e** qPCR analysis of CG18609 and CG3739 showing mRNA levels normalized to *actin5c* expression. Each dot represents a biological replicate and lines indicate means ± SEM. *p* values are based on ANOVA with Sidak’s posthoc test. *n* = 3 per group

**Fig. 7:**
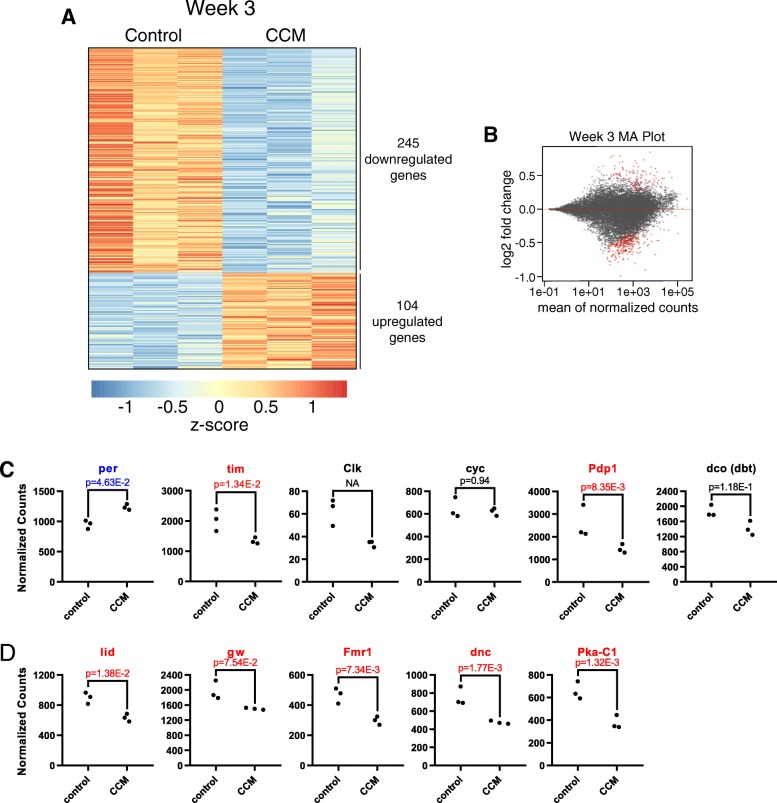
Large-scale changes in gene expression following 3 weeks of CCM exposure. **a** Heatmap showing z-score of DEseq2 normalized counts for all genes found to be differentially expressed (at a FDR < 0.1) following 3 weeks of control or CCM conditions. Each condition and time point includes 3 biological replicates. **b** MA plot for week 3 differential expression analysis. Log2 fold change (M) is plotted against average normalized gene expression (A). Red dots indicate differentially expressed genes. (**c**) Expression plots showing normalized gene expression levels of canonical core clock genes following 3 weeks of CCM or control conditions. Significantly upregulated genes (at a FDR < 0.1) are in blue and significantly downregulated genes are in red. Dots represent individual samples. (**d**) Expression plots of genes implicated in circadian rhythm regulation are graphed as in C. All genes in (**d**) were significantly downregulated by CCM

We began by monitoring transcriptional changes following 2 weeks of CCM exposure. Somewhat surprisingly, only 7 genes were found to display differential expression at this time, with 6 downregulated and 1 upregulated by CCM (Fig. 6a-d and Additional file 1). We then used a similar analysis to compare gene expression levels following 3 weeks of exposure to control or CCM conditions. This analysis identified 349 genes exhibiting differential expression, with 245 downregulated and 104 upregulated by CCM (Fig. 7a-d and Additional file 2). Importantly, qPCR analysis on a separate cohort of flies confirmed CCM-induced expression changes for several candidate genes (Fig. 6e). Finally, to compare the effects of chronic CCM exposure with those associated with normal aging, we also conducted a differential expression analysis with samples obtained following 2 or 3 weeks of exposure to control conditions. We found that 4159 genes were differentially expressed between these two time points, with 2349 downregulated and 1810 upregulated in week 3 control samples compared to week 2 (Additional file 3). These represent genes that are regulated by aging independent of CCM exposure.

**Fig. 8:**
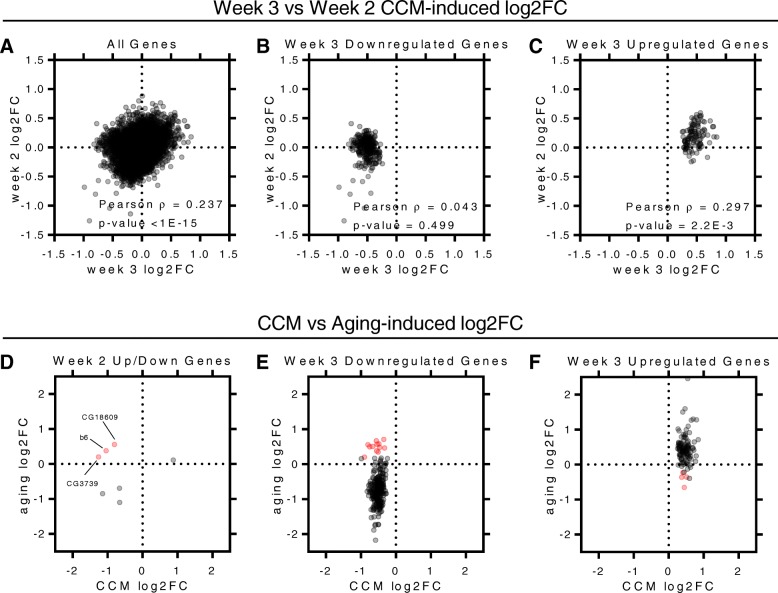
CCM-induced gene expression changes mirror those that occur during aging. (**a**-**c**) Scatterplots with week 3 CCM-induced log2FC graphed on the x-axis and week 2 CCM-induced log2FC graphed on the y-axis for all genes (**a**), week 3 downregulated genes (**b**) and week 3 upregulated genes (**c**). Each dot represented an individual gene. For all genes (**a**) *n* = 14,062; Spearman rho = 0.237 (95% confidence interval: 0.221 to 0.252); *p* < 1.0E-15. For week 3 downregulated genes (**b**) *n* = 245; Spearman rho = 0.043 (95% confidence interval: − 0.082 to 0.168); *p* = 0.499. For week 3 upregulated genes (**c**) *n* = 104; Spearman rho = 0.297 (95% confidence interval: 0.111 to 0.463); *p* = 2.20E-3. (**d**-**f**) Scatterplots with week 2 or 3 CCM-induced log2FC graphed on the x-axis and aging-induced log2FC graphed on the y-axis for all week 2 differentially expressed genes (**d**), week 3 downregulated genes (**e**), and week 3 upregulated genes (**f**). Each dot represents an individual gene. Red dots indicate genes that are oppositely regulated by CCM and aging. See Table 5 for a list of these oppositely regulated genes

On a global level, we found that changes in gene expression in week 2 were positively correlated with week 3 changes (Fig. 8a). This suggests that the gene expression changes induced by circadian misalignment are persistent and progressive, although it should be stressed that the correlation between week 2 and week 3 changes was relatively weak and did not hold for all genes. Interestingly, this correlative relationship persisted when we specifically looked at week 3 upregulated genes, but was not present among downregulated genes (Fig. 8b and c). Furthermore, the vast majority of week 3 upregulated genes had positive log2FC in week 2 (Fig. 8c). This demonstrates that genes that are strongly upregulated following 3 weeks of CCM exposure are already showing signs of upregulation by week 2, though these changes are not statistically significant at the 2-week time point.

Although expression changes in week 3 downregulated genes as a whole were not correlated with week 2 changes, there were several individual genes that were similarly affected following different CCM exposure times. Notably, 3/6 genes found to be downregulated following 2 weeks of CCM exposure were also identified as significantly differentially expressed in week 3, providing independent confirmation of these changes. The other 3 week 2 downregulated genes showed a trend towards decreased expression in week 3 samples; however, these genes also showed substantial age-related decreases in expression in control flies that somewhat mitigated the expression differences between control and CCM flies at the week 3 time point (Fig. 6c and d).

### CCM-induced gene expression changes indicate cellular stress and organismal decline

To identify functional gene categories that were overrepresented among differentially expressed genes, we performed Gene Ontology (GO) term analysis. Although only a handful of genes were found to be differentially expressed following 2 weeks of CCM exposure, genes involved in lipid metabolism were overrepresented among this dataset, as 3/6 downregulated genes were annotated with this function (Table 2, Fig. 6c and Additional file 4). These included CG4830, which is predicted to act as a fatty acid ligase, CG18609, which is predicted to act as a fatty acid elongase important for the synthesis of very long chain fatty acids, and Acox57D-d, which is an acyl-CoA oxidase that contributes to lipid homeostasis. Although these genes are as of yet poorly characterized, their shared function in regulating lipid metabolism highlights a potentially important role of altered lipid homeostasis as an early mediator of the negative health consequences of CCM, especially as these week 2 gene expression changes occur prior to most CCM-induced death (Fig. 1). Notably, we found that CG18609 remained downregulated following 3 weeks of CCM exposure, while CG4830 and Acox57D-d were both strongly downregulated by aging such that expression levels in control flies were indistinguishable from CCM flies at this later time point (Fig. 6c and Additional file 1). Thus, for these two genes, it appears that CCM accelerates the age-related decline in expression, while for CG18609 the impact of CCM counteracts the normal aging-related increase in expression.

**Table 2:** Overrepresented GO Terms Associated with Week 2 Differentially Expressed Genes

GO ID	GO Term	Adjusted *p*-Value	Number of Genes	Fold Enrichment
GO:0006631	fatty acid metabolic process	3.00E-04	3	58.40
GO:0006082	organic acid metabolic process	1.38E-02	3	16.13
GO:0006629	lipid metabolic process	2.01E-02	3	14.18
GO:0016053	organic acid biosynthetic process	3.84E-02	2	32.40

Adjusted *p*-value is based on Bonferroni correction. Number of genes refers to genes annotated with that term out of the 6 week 2 downregulated genes. Fold enrichment is compared to the relative prevalence of genes annotated with the term in the full set of *Drosophila* coding genes.

GO term analysis for week 3 differentially expressed genes revealed 377 GO terms associated with week 3 downregulated genes, and these prominently included terms associated with developmental processes, regulation of gene expression, and biosynthetic pathways (Table 3 and Additional file 5). We also found several terms related to nervous system structure and function, including nervous system development, synapse assembly and organization, synaptic transmission, and behavioral processes such as learning and memory and generation of locomotor rhythms (Table 4). This latter category is of particular interest, and we found that CCM altered expression both of canonical core clock genes (Fig. 7c) as well as other genes that have been implicated in circadian regulation (Fig. 7d). Interestingly, among core clock genes, we found that *timeless* and *Pdp1* were significantly downregulated while *period* was upregulated by CCM. These changes mirror those we and others have observed with normal aging (Additional Fig. 3) [25]. It is interesting that circadian misalignment alters the expression of circadian regulatory genes, despite the fact that our DD behavioral analysis failed to provide evidence for a damaged or dysfunctional molecular clock. One possibility to explain this discrepancy could be that peripheral clocks are more strongly affected by CCM, since our sequencing data was conducted on whole flies and rest:activity rhythms are mainly under the control of central brain clocks. It is also possible that changes in gene expression were too subtle to affect free-running rhythms, especially since central circadian clock function is robust to perturbations in clock gene expression [26].

**Table 3:** Overrepresented GO Terms Associated with Week 3 Differentially Expressed Genes

GO ID	GO Term	Adjusted *p*-Value	Number of Genes	Fold Enrichment
**--**Terms Associated with Downregulated Genes--				
GO:0048869	cellular developmental process	5.90E-34	109	3.6
GO:0048468	cell development	1.02E-32	97	4.0
GO:0030154	cell differentiation	1.13E-32	106	3.6
GO:0007275	multicellular organism development	1.02E-31	123	3.0
GO:0048731	system development	7.36E-29	102	3.4
GO:0048513	animal organ development	1.39E-21	76	3.7
GO:0007399	nervous system development	2.15E-21	72	3.9
GO:0010468	regulation of gene expression	7.02E-21	84	3.3
GO:0009888	tissue development	4.72E-20	69	3.8
GO:0032989	cellular component morphogenesis	1.11E-19	55	4.8
GO:0022008	neurogenesis	1.51E-19	62	4.2
GO:0009790	embryo development	7.70E-19	49	5.2
GO:0009889	regulation of biosynthetic process	7.12E-18	74	3.3
GO:0007389	pattern specification process	7.83E-18	46	5.3
GO:0051128	regulation of cellular component organization	2.56E-17	56	4.2
**--**Terms Associated with Upregulated Genes--				
GO:0009636	response to toxic substance	5.59E-05	10	9.0
GO:0051186	cofactor metabolic process	1.13E-04	12	6.5
GO:0006790	sulfur compound metabolic process	1.06E-03	9	7.6
GO:0061077	chaperone-mediated protein folding	6.69E-03	5	15.2
GO:0008340	determination of adult lifespan	9.98E-03	8	6.6
GO:0051085	chaperone cofactor-dependent protein refolding	1.02E-02	4	22.3
GO:0046680	response to DDT	1.21E-02	3	44.6
GO:0007568	aging	1.41E-02	8	6.3
GO:0009408	response to heat	1.83E-02	6	9.1
GO:0006979	response to oxidative stress	2.14E-02	7	7.1
GO:0055114	oxidation-reduction process	3.19E-02	14	3.3
GO:0009266	response to temperature stimulus	3.71E-02	7	6.5
GO:0034620	cellular response to unfolded protein	4.73E-02	4	15.3

Adjusted *p*-value is based on Bonferroni correction. Number of genes refers to genes annotated with that term out of the 245 week 3 downregulated genes or 104 week 3 upregulated genes. Fold enrichment is compared to the relative prevalence of genes annotated with the term in the full set of *Drosophila* coding genes.

**Table 4:** Overrepresented Nervous System Related GO Terms Associated with Week 3 Downregulated Genes

GO ID	GO Term	Adjusted *p*-Value	Number of Genes	Fold Enrichment
GO:0007399	nervous system development	2.15E-21	72	3.9
GO:0022008	neurogenesis	1.51E-19	62	4.2
GO:0061564	axon development	9.25E-11	30	5.3
GO:0050808	synapse organization	1.13E-06	23	4.7
GO:0007611	learning or memory	3.34E-06	17	6.2
GO:0050890	cognition	3.34E-06	17	6.2
GO:0007420	brain development	1.27E-04	13	6.6
GO:0040040	thermosensory behavior	6.09E-04	6	19.3
GO:0007416	synapse assembly	1.61E-03	14	4.9
GO:0045475	locomotor rhythm	4.24E-03	9	7.6
GO:0007417	central nervous system development	5.05E-03	17	3.7
GO:0007268	chemical synaptic transmission	1.34E-02	17	3.5

Adjusted *p*-value is based on Bonferroni correction. Number of genes refers to genes annotated with that term out of the 245 week 3 downregulated genes. Fold enrichment is compared to the relative prevalence of genes annotated with the term in the full set of *Drosophila* coding genes.

GO term analysis of week 3 upregulated genes suggested that CCM-exposed flies were experiencing high levels of cellular stress. We identified 17 overrepresented GO terms associated with week 3 upregulated genes, and these included functions such as response to toxic substances, unfolded protein response, response to oxidative stress, and determination of lifespan (Table 3 and Additional file 6). These findings demonstrating increased expression of stress-related genes, together with the downregulation of genes involved in developmental and biosynthetic processes, are consistent with the idea that CCM accelerates organismal decline.

In line with this, we noted that the majority of differentially expressed genes following 3 weeks of CCM exposure were also found to be similarly regulated by aging in control samples. Thus, 145/245 CCM downregulated genes and 50/104 CCM upregulated genes were similarly affected by aging (using a Benjamini-Hochberg FDR cutoff of 0.1 to identify genes differentially expressed with aging). Furthermore, an additional 50 CCM downregulated genes and 22 CCM upregulated showed a trend towards aging-related changes in the same direction (genes in which aging-related log2FC was in the same direction as the CCM-induced log2FC and had a Benjamini-Hochberg adjusted *p*-value between 0.1 and 0.3) (Additional file 2). This indicates that CCM may accelerate or exacerbate normal age-related changes in gene expression.

CCM-induced gene expression changes could directly result from circadian misalignment or could simply reflect the negative health state of the animal, especially after 3 weeks of exposure to aberrant lighting schedules. For genes that are similarly regulated by these two processes, it is difficult to differentiate between these possibilities. It is undoubtedly the case that some genes are convergently regulated by CCM and aging and therefore that genes that are similarly affected by these two processes may be important and direct contributors to the effects of CCM. However, we were particularly interested in finding genes that were specifically regulated by CCM with respect to aging, because these gene expression changes could be unequivocally identified as direct consequences of CCM. We therefore searched for genes that had statistically significant changes in gene expression due to CCM that exhibited changes in the opposite direction with aging. We used a somewhat relaxed FDR cutoff of 0.3 for aging-related changes in expression in this case, which we reasoned would allow us to pinpoint genes that were either oppositely regulated by CCM and aging or, at the very least, were significantly altered by CCM and unchanged by aging. Notably, only 12 CCM downregulated genes and 4 CCM upregulated genes satisfied these criteria (Table 5). Significantly, analysis of the oppositely regulated genes demonstrated an overrepresentation of genes involved in lipid metabolism as well as transmembrane transport. The overrepresentation of lipid metabolism genes again highlights alterations in this functional category as possibly mediating the negative health consequences of CCM. Among these genes, the fatty acid elongase CG18609 was found to be significantly downregulated following both 2 and 3 weeks of CCM and to be significantly upregulated during normal aging (Table 5). Thus, its function in lipid metabolism combined with its gene expression profile make CG18609 an attractive candidate for future investigations into the negative effects of CCM.

**Table 5:** Genes Oppositely Regulated by Circadian Misalignment and Aging

gene name	week 3 log2FC	week 3 *p*-adj	aging log2FC	aging *p*-adj	function
--Downregulated with CCM; Upregulated with Aging--					
*CG3739*	*−0.90*	*3.70E-13*	*0.20*	*2.38E-01*	*dipeptidyl-peptidase activity; serine-type peptidase activity*
*CG18609*	*−0.80*	*7.72E-13*	*0.56*	*4.94E-04*	*fatty acid elongase activity*
CG34180	−0.75	1.46E-03	0.50	1.04E-01	unknown
CG10960	−0.67	1.49E-06	0.51	4.81E-03	carbohydrate:proton symporter activity; glucose transmembrane transporter activity
CG11741	−0.56	4.57E-02	0.67	3.26E-02	unknown
*b6*	*−0.55*	*4.23E-02*	*0.38*	*1.79E-01*	*unknown*
Uro	−0.54	2.75E-02	0.57	3.79E-02	urate oxidase activity
rost	−0.51	2.96E-02	0.35	1.14E-01	unknown
CG4607	−0.48	7.35E-03	0.58	1.96E-04	carbohydrate:proton symporter activity; glucose transmembrane transporter activity
CG15096	−0.46	9.90E-02	0.48	4.42E-02	transmembrane transporter activity
CG9459	−0.35	8.04E-02	0.71	1.34E-03	fatty acid elongase activity
CG14464	−0.33	9.77E-02	0.46	1.09E-02	unknown
--Upregulated with CCM; Downregulated with Aging--					
CG5804	0.51	7.70E-03	−0.36	1.68E-01	fatty-acyl-CoA binding
CG5767	0.45	4.87E-02	−0.65	6.97E-03	unknown
CG34330	0.41	3.14E-02	−0.22	2.57E-01	unknown
AsnS	0.37	7.58E-02	−0.36	8.08E-02	asparagine synthase (glutamine-hydrolyzing) activity; protein homodimerization activity

log2FC determined by DEseq2 using shrinkage estimation. *p*-adj is the False Discovery Rate (FDR) as determined by Benjamini-Hochberg correction. Italicized genes were also significantly downregulated following 2 weeks of CCM exposure.

## Discussion

We have chronically exposed flies to aberrant lighting schedules in order to better characterize the behavioral, physiological and molecular consequences of circadian misalignment. Our findings of reduced longevity demonstrate the profound consequences of CCM, and are in line with a number of other studies conducted in flies and mammals. In one of the earliest studies to test the functional significance of circadian rhythms in animals, Pittendrigh and Minis demonstrated that exposure of fruit flies to long (27-h) or short (21-h) days (the equivalent of daily 3-h phase delays or advances) resulted in reduced lifespan [10], and these findings were later corroborated by similar studies in blowflies [11]. In mammalian models, as in invertebrate systems, studies have consistently demonstrated negative health consequences of CCM, particularly for aged or disease-prone animals. Chronic misalignment has been found to increase mortality in aged mice [14], immune-challenged mice [13] and mice and hamsters prone to cardiovascular disease [15] and cancer [17]. In addition to these studies demonstrating that CCM can exacerbate or hasten disease progression, misalignment has also been found to decrease longevity in otherwise healthy mammals. For example, reduced lifespan was observed in mice exposed to an extremely short 8-h light-dark cycle, an effect that was absent in mutant mice that lacked a functional molecular clock [16]. More recently, it was demonstrated that wildtype mice exposed to weekly phase shifts exhibited signs of neurodegeneration, kidney disease, ulcerative dermatitis, and cancer, and these were associated with reduced lifespan [9]. It should be noted that CCM paradigms in mammals have typically involved less frequent phase shifts than paradigms used in flies. It is likely that the severity of the consequences of CCM will vary depending on the extent and duration of misalignment.

The effects of exposing organisms to non-24-h light cycles provided the basis for Pittendrigh’s theory of circadian resonance, which stated that organisms function best when the difference between endogenous circadian period and environmental cycles is small, thus minimizing the magnitude of daily circadian clock adjustments [10]. Other studies have directly tested the resonance theory by showing that the negative consequences of exposing animals to non-standard length days were at least partially ameliorated in mutant animals whose endogenous period matched that of the aberrant lighting schedule [12, 27–29]. In general, these studies demonstrate that large daily adjustments to the circadian clock are detrimental to organismal health.

CCM could negatively influence organismal health for several reasons, which are not mutually exclusive. First, misalignment could result in a loss of the extrinsic advantage of circadian rhythms to properly time behavioral and physiological processes with respect to external environmental conditions. This possibility is supported by studies of wild-caught chipmunks, in which surgical destruction of the central circadian clock led to premature death, likely due to mistimed activity leading to higher rates of predation [30]. However, in controlled laboratory experiments such as those we have conducted, which include ad libitum food availability and a lack of predation, it is unlikely that extrinsic factors play a prominent role in producing adverse health effects such as decreased longevity. One exception, however, is that some physiological processes may be optimized to occur in either the light or dark. If this were the case, then we would expect our CCM flies to be negatively affected since they show evidence of mistimed behavior, particularly in the hours preceding lighting transitions, which suggests that other physiological processes may be similarly mistimed.

A second possibility is that CCM causes internal desynchrony such that physiological and behavioral processes become disordered with respect to one another. This is thought to be a major cause of jetlag symptoms in mammals, as studies have shown that clocks in different tissues resynchronize to phase shifts at different rates [31]. Furthermore, studies in humans have shown that gene expression rhythms in peripheral tissues quickly dampen under conditions of imposed circadian misalignment, likely due to a loss of synchronization between behavioral schedules and the central circadian clock [32, 33]. It is unclear to what extent this type of internal desynchrony occurs in flies. Our locomotor activity tracking demonstrated apparent entrainment of rest:activity rhythms to the 28-h day produced by daily 4-h phase delays; however, further studies are necessary to assay clock protein cycling in central and peripheral tissues under these conditions to determine whether molecular clocks are indeed entrained to long or short environmental cycles and to test whether central and peripheral oscillators remain synchronized to one another. Even if entrainment is occurring, the substantial discrepancy between the 28-h environmental cycles and the ~ 24-h endogenous period of the fly may be challenging the efficiency of the endogenous clock in CCM flies, as substantial daily adjustment to the phase of the clock would be required. Furthermore, CCM conditions could still result in transient desynchrony occurring on a daily basis, especially towards the end of the extended dark period because clock cycling cannot be adjusted by entrainment mechanisms until subsequent light exposure.

Circadian misalignment could also indirectly affect health secondary to changes in overall activity or sleep levels, which are known to accompany CCM [4, 7, 8]. In general, we found only very modest effects of CCM on sleep duration in flies. Importantly, there was no correlation between sleep amount and longevity in female flies, which indicates that the slightly reduced sleep exhibited by CCM females in the first week of the experiment could not account for their reduced longevity. Furthermore, although sleep amount and longevity were correlated in males, multivariate models demonstrated that CCM decreased longevity to a further extent than what would be expected based on the effect of CCM on sleep. Thus, while sleep disturbances undoubtedly contribute to the negative consequences associated with CCM in humans, our results clearly demonstrate that CCM can reduce lifespan independent of its effects on sleep.

To better understand the proximal causes of the adverse health effects of CCM, we conducted RNA sequencing of flies following 2 or 3 weeks of circadian misalignment. Notably, the 2-week time point occurs prior to most CCM-induced death, which we reasoned would allow us to identify early molecular changes directly associated with CCM as opposed to nonspecific changes occurring as a consequence of CCM-induced organismal decline. Consistent with the relatively healthy state of CCM-exposed flies at 2 weeks, we found few genes that exhibited significant up- or downregulation at this time. Nevertheless, analysis of differentially expressed genes demonstrated an overrepresentation of those associated with lipid metabolism. The relatively early expression changes in these genes suggests that alterations in lipid metabolism could underlie the initial consequences of CCM.

In addition to investigating gene expression changes associated with CCM, we analyzed control samples to identify genes regulated by aging independent of circadian misalignment. Notably, we found that many more genes were differentially expressed in response to aging compared to CCM. The aging-related genes we observed included many known to be upregulated with aging. For example, we found that the heat-shock genes *hsp22* and *hsp70* were upregulated with aging and were both among the top 5 differentially expressed genes in our analysis. These genes have been found previously to be robustly upregulated with aging in fly heads and whole flies [25, 34].

In order to identify gene expression changes specific to CCM exposure, we isolated genes in which the consequences of CCM were opposite those of aging. Interestingly, genes involved in lipid binding and metabolism were also overrepresented among this dataset, further pointing towards an important contribution of altered lipid homeostasis in mediating the negative effects of CCM. Given the wide range of functions associated with lipid metabolism, more focused experiments will be necessary to determine the specific contribution of the genes implicated in our studies to the negative consequences of CCM. However, it is of note that several candidate genes are involved in fatty acid elongation, which is essential for the creation of very long-chain fatty acids and sphingolipids that make up integral parts of cell membranes and have been implicated in diverse processes including cell growth and cell signaling [35]. This includes CG18609, which is a fatty acid elongase that we found to be significantly downregulated following both 2 and 3 weeks of CCM. CG18609 normally increases in expression as flies age, which indicates that the gene expression changes are specific to CCM and are not simply occurring alongside CCM-induced organismal decline. Interestingly, lipid metabolism has been linked to aging processes [36]. Furthermore, genes involved in lipid metabolism were also recently found to be acutely dysregulated in human skeletal muscle following a 12-h phase shift [37] as well as in livers of mice exposed to weekly 8-h phase shifts [9], which suggests that lipid metabolism may be affected by CCM across multiple species.

In order to control for circadian variation in gene expression, we extracted our RNA samples between ZT0–3 in both CCM and control flies. At this time, most flies in both groups are awake and active. However, we cannot rule out that some gene expression changes observed in CCM flies reflect the increased activity in the preceding few hours due to the prolonged morning anticipation in these flies, or to potential differences in feeding behavior associated with this prolonged activity. An assessment of gene expression in CCM flies across multiple circadian time points and behavioral states would help to differentiate between these possibilities.

Many more genes exhibited differential expression following 3 weeks of CCM compared to only 2 weeks. This difference may be due to a progressive dysregulation of expression such that changes are underway by week 2 that are too subtle to detect with whole fly RNA sequencing. The fact that there was an overall correlation between gene expression changes in weeks 2 and 3 supports this idea. On the other hand, it is possible that many of the gene expression changes observed in week 3 reflect general organismal decline, which appears to be accelerated in CCM-exposed flies. In fact, we saw substantial overlap between genes altered by CCM and those that exhibited expression changes due to aging. These included upregulation of stress-response genes and downregulation of genes involved in development and regulation of gene expression. In addition, we observed dysregulation of a number of genes involved in neuronal function, including those responsible for generation of circadian rhythms. This may indicate that molecular clock function is compromised, which could in turn exacerbate the negative health consequences of CCM, especially as the circadian clock protects against oxidative stress in aged flies [25, 38]. However, given that we found no effect of CCM on free-running behavioral rhythms, which are controlled by the central clock, our results are more consistent with reduced circadian function of peripheral clocks. Tissue-specific RNA sequencing in the face of circadian misalignment would help to determine whether this is the case. If peripheral clock function is indeed altered, then the reduced longevity produced by CCM could be a consequence both of the acute physiological effects of misalignment and a reduced ability to effectively mitigate those effects.

## Conclusions

Our results confirm that CCM is associated with severe, adverse consequences, including reduced lifespan. We furthermore show that the negative health effects of CCM can occur independent of changes in sleep amount, which indicates that frequent circadian phase shifts are inherently unhealthy, likely due to behavioral and physiological processes occurring at sub-optimal times. Finally, our RNA sequencing analysis demonstrates that CCM produces large-scale changes in gene expression, and in particular identifies genes involved in lipid metabolism as being potentially important mediators of the negative health consequences of CCM. These studies identify candidate molecules and pathways that can be targeted in future studies to further delineate the etiology of CCM-induced organismal decline. Our findings have important implications for the large number of people who routinely follow behavioral schedules that run outside the dictates of their internal circadian clocks.

## Methods

### Longevity analysis

Prior to behavioral experiments, fly stocks were maintained in 6 oz. square-bottom polypropylene bottles (Fisher Scientific) with standard cornmeal-molasses food on a 12:12 light:dark (LD) cycle at 25 °C. For longevity experiments, male and female iso31 flies [39] were collected under light CO_2_ anesthesia within 1 day of eclosion. Individual flies were loaded into glass tubes containing a 5% sucrose and 2% agar food source for locomotor activity analysis with the Drosophila Activity Monitoring (DAM) system (Trikinetics, Waltham, MA). Humidity and temperature-controlled incubators (Percival, Perry IA) were used to expose flies to either a 24-h schedule (LD 12:12; control group), or a 28-h schedule (LD 14:14; CCM group), which results in daily 4-h phase delays. A 28-h schedule was chosen because previous studies indicated a more severe consequence of long versus short days on Drosophila lifespan [10] and because a daily 4-h phase shift results in CCM-exposed flies completing 6 full LD cycles in a week such that total weekly light and dark exposure was equivalent between CCM and control flies. Incubator temperature was held constant at 25 °C and humidity levels were kept between 70 and 80%. Temperature, humidity and light intensity were checked regularly to ensure uniformity between experimental groups. Flies were transferred to new tubes each week to supply fresh food. Occasionally, flies escaped during transfer to new tubes, and these flies were excluded from all analyses. Longevity analysis was conducted in two independent experiments, each of which included 64 male and female flies for each condition. Because consistent results were observed between these two experiments, we combined data for analysis.

The DAM system records each time a fly breaks an infrared beam that bisects the center of each glass tube. Longevity was determined by identifying the last minute that a beam break occurred. Occasionally we observed “ghost” readings, where single beam breaks were detected after extended periods of inactivity. Thus, we ignored isolated activity bins that occurred > 12 h after previous activity. Data were collected until all flies in the experiment were dead. Fly lifespans were analyzed with the Log-rank test (GraphPad Prism 7), with *p* < 0.05 considered significant.

### Locomotor activity and sleep analysis

Analysis of locomotor activity rhythmicity was performed with ClockLab software (Actimetrics, Wilmette, IL). Locomotor activity rhythm period and strength (power) were determined with chi^2^ periodogram analysis. Rhythm power was defined as the amplitude of the periodogram line at the dominant period minus the chi^2^ significance line at a significance of *p* < 0.01. Weekly LD rhythmicity was concurrently determined for the same group of flies that were used for longevity experiments. In a separate group of flies, the period and power of free-running rhythms under conditions of constant darkness (DD) were calculated to assess the health of the endogenous circadian clock. These flies were exposed to control or CCM conditions for 1–3 weeks and then transferred to DD for an additional week for rhythmicity analysis. Because fly activity is affected by transfer between LD and DD incubators, the first day of DD was not included in rhythmicity analysis. For all rhythmicity analyses the bin length was 1 min. 2-Way ANOVA followed by Sidak’s posthoc test (GraphPad Prism 7) were used to compare rhythm strength between control and CCM flies at the different time points, with *p* < 0.05 considered significant.

The average (normalized) function in ClockLab was used to create mean normalized activity plots in Fig. 3i. Flies were kept in LD 14:14 (CCM) or 12:12 (control) conditions for 2 weeks prior to transfer to DD, and graphs show the final 3 days in LD followed by the first 3 days in DD.

Sleep was defined as 5 consecutive minutes of inactivity [40] as determined by a custom-developed Excel formula. 2-Way ANOVA followed by Sidak’s posthoc test (GraphPad Prism 7) were used to separately compare activity (in mean beam breaks/min) and sleep amount (in mean min/hr) between control and CCM flies for week 1, week 2, week 3, and full-life, with p < 0.05 considered significant. For full-life measurements, we removed the last three days of life from analysis because flies exhibited reduced activity during this time, making it difficult to separate sleep from an aging-induced decreases in locomotor activity.

For weekly rest:activity rhythm period and power as well as activity amount and sleep duration analysis, only flies that survived through the entire week were included and thus the number of flies analyzed varied between weeks. For female control flies, 121 flies survived through week 1, 118 survived through week 2, 92 through week 3, and 26 through week 4. For female CCM flies, 123 survived through week 1, 119 through week 2, 52 through week 3, and 13 through week 4. For control male flies, 125 survived through week 1, 124 through week 2, 84 through week 3, and 27 through week 4. For CCM male flies, 115 survived through week 1, 107 through week 2, 44 through week 3, and 6 through week 4. Thus, these are the ns for experiments depicted in Figs. 3 and 4. For Fig. 2, ns are slightly smaller since analysis was conducted starting from ZT18 on day 1 to ZT18 of day 8 for each week instead of starting at ZT0, and a handful of flies died between ZT0 and ZT18 at the end of the week.

Spearman’s rank test (GraphPad Prism 7) was used to calculate the correlation between sleep and longevity, with *p* < 0.05 considered significant. To further assess the effect of CCM and sleep time on longevity the Cox Proportional Hazard model was used. Hazard ratios for CCM and sleep time were first calculated in univariate models. For male flies, a multivariate Cox Proportional Hazard Model was also used with both treatment and week 1 sleep time in the model to determine if CCM reduced lifespan independent of its effects on sleep duration. In a separate approach, each control fly was matched with a CCM-exposed fly with a similar sleep time (within 0.5 min/hr) and a hazard ratio for CCM was calculated in this smaller data set.

### RNA sequencing

Male and female iso31 flies were exposed to 2 or 3 weeks of control or CCM conditions in DAM monitors as described above. 10 male and 10 female flies were collected for each sample (3 CCM and 3 control samples at each time point, 12 total) and total RNA was extracted at ZT0–3 with TRIreagent (Molecular Research Center, Inc., Cincinnati, OH) according to the manufacturer’s recommendations. Following phase separation, samples were additionally subjected to DNAse treatment and purification with the Zymo Direct-zol RNA kit (Zymo Research, Irvine, CA). All samples passed quality control with RIN > 7.2 and a 260/280 ratio > 2.0. Library preparation with the NEB Next Ultra II kit and subsequent 150 base pair, paired-end RNA sequencing on the Hi-Seq platform were conducted by Novogene (Davis, CA).

Approximately 21–28 million raw reads were obtained per sample. Novogene filtered raw reads to remove reads containing adapters, reads containing *N* > 10% (N represents the bases that cannot be determined), and reads containing Phred quality score ≥ 5 over more than 50% of the read. Following filtering, > 94.78% of reads remained for each sample.

### Differential gene expression analysis

Reads were mapped to Dmel Reference Genome (dm6) with RNA Star [41] in Galaxy (using the public Galaxy server at usegalaxy.org). Drosophila_melanogaster.BDGP6.87.gtf was used for the annotation file and filtered to remove all non-coding genes because we were interested in identifying differentially expressed genes that encode for proteins. The aligned BAM files were processed with MMquant [42] to quantify the number of reads that mapped to each gene. MMquant allows for quantification of duplicated genes by creating merged genes out of different genes for which the sequence is multiply mapped.

DEseq2 [43] in Galaxy was used to identify genes exhibiting differential expression between CCM and control samples at the 2- and 3-week time points. We used a Benjamini-Hochberg FDR cutoff of 0.1 to determine differentially expressed genes, but used no cutoff for fold-change (FC), which was generally in the moderate range (log2FC ranged from − 1.26 to 0.88). DEseq2 uses shrinkage estimate to generate a conservative estimate of FC.

We also used DEseq2 to assess aging-related changes by comparing gene expression in control samples between week 2 and 3. We then identified genes among the 349 that were found to be up- or downregulated following 3 weeks of CCM that were oppositely regulated by aging, which included those with a log2FC in the opposite direction as that produced by CCM and that were significant with a FDR cutoff of 0.3.

We used the Pearson correlation coefficient (GraphPad Prism 7) to assess the correlation between the CCM-induced log2FC in gene expression between week 2 and week 3 for all genes as well as the subsets of genes found to be up- or downregulated following 3 weeks of CCM. *p* < 0.05 was considered significant.

### Gene ontology (GO) term analysis

GO term analysis for Biological Function was performed with Princeton University’s GOTermFinder (http://go.princeton.edu/cgi-bin/GOTermFinder). Analysis was done separately for up- and down-regulated genes from each week. Because some genes have unique as well as overlapping sequences and therefore showed up more than once in the MMquant output, we manually curated the differentially expressed gene list to include only one incidence of each gene for gene ontology analysis. We provided the background gene list which consisted of all genes tested for differential expression. The Bonferroni-corrected *p*-value cutoff was set to 0.05 to determine over- or underrepresented GO terms. The resulting lists were then passed through REVIGO (http://revigo.irb.hr) [44] with an allowed similarity of 0.7 to remove redundant terms. Because there were > 350 GO terms associated with week 3 downregulated genes, we further filtered that list to remove highly generic terms (those with an average distance to root of < 4). Table 3 lists only the top 15 most significant of these filtered GO terms.

### Quantitative PCR (qPCR) analysis

Male and female iso31 flies were exposed to 2 or 3 weeks of control or CCM conditions in DAM monitors and RNA extraction was performed as described above. Flies used for qPCR analysis were from a separate cohort from those used for RNA sequencing. cDNA synthesis was conducted with the High Capacity cDNA Reverse Transcription Kit (Applied Biosystems) from 1 μg total RNA and qPCR was conducted with the PowerUp SYBR Green Master Mix (Applied Biosystems) using a StepOne Plus Real-Time PCR System (Applied Biosystems). We included 3 samples per group and 3 technical replicates per sample. Primers were as follows: CG18609 forward: 5′- CTCTTCATCGTGGGCATTTGC – 3′; CG18609 reverse: 5′- TCGAGGACCTTGTTCAGCAG -3′; CG3739 forward: 5′- AGGATAGTGACTTGCCCTGG – 3′; CG3739 reverse: 5′- GTGGATCCAAAGGGTTGGCT -3′. Quantification was done with the relative standard curve method, and gene expression was normalized to *actin5c* levels for each sample. RNA sequencing showed no expression changes in *actin5c* with CCM or aging. ANOVA with Sidak’s posthoc test was used to compare normalized gene expression levels. *p* < 0.05 was considered significant.

## Additional files

Additional file 1: Week 2 differentially expressed genes. List of all differentially expressed genes following 2 weeks of CCM exposure (at FDR < 0.10). (XLSX 13 kb)

Additional file 2: Week 3 differentially expressed genes. List of all differentially expressed genes following 3 weeks of CCM exposure (at FDR < 0.10). (XLSX 114 kb)

Additional file 3: Aging differentially expressed genes. List of all differentially expressed genes following 3 weeks compared to 2 weeks of control conditions (at FDR < 0.10). (XLSX 888 kb)

Additional file 4: GO term analysis of week 2 downregulated genes. List of all overrepresented GO terms associated with week 2 downregulated genes (at Bonferroni-adjusted *p* < 0.05). (XLSX 10 kb)

Additional file 5: GO term analysis of week 3 downregulated genes. List of all overrepresented GO terms associated with week 3 downregulated genes (at Bonferroni-adjusted *p* < 0.05). (XLSX 61 kb)

Additional file 6: GO term analysis of week 3 upregulated genes. List of all overrepresented GO terms associated with week 3 upregulated genes (at Bonferroni-adjusted *p* < 0.05). (XLSX 11 kb)
